# Consumption of fast foods and ultra-processed foods and breast cancer risk: a systematic review and meta-analysis

**DOI:** 10.1186/s41256-025-00425-x

**Published:** 2025-07-01

**Authors:** Mehdi Karimi, Reyhaneh Rabiee, Farnaz Hooshmand, Baharak Aghapour, Mina Ahmadzadeh, Elahe Havaei, Kimia Kazemi

**Affiliations:** 1 Faculty of Medicine, Bogomolets National Medical University (NMU), Kyiv, Ukraine; 2 Department of Nutrition, School of Public Health, Shahid Sadoughi University of Medical Sciences, Yazd, Iran; 3 Faculty of Medicine, Golestan University of Medical Science, Gorgan, Iran; 4 Department of Community Nutrition, School of Nutrition and Food Sciences, Tabriz University of Medical Sciences, Tabriz, Iran; 5 Department of Nutrition and Dietetics, Shahid Beheshti University of Medical Sciences (SBUMS), Tehran, Iran; 6 Islamic Azad University, Central Tehran Branch, Tehran, Iran; 7 Department of Food Science and Technology, Islamic Azad University, Ayatollah Amoli Branch, Amol, Iran

**Keywords:** Processed food, Fast food, Breast cancer, Risk, Nutrition, Epidemiology

## Abstract

**Background:**

The increasing consumption of fast foods (FFs) and ultra-processed foods (UPFs) worldwide has raised concerns due to their association with carcinogenic compounds and potential links to various cancers. However, this evidence about breast cancer risk remains inconsistent. This study aimed to meta-analyze the association between FFs and UPFs consumption and the risk of breast cancer in females.

**Methods:**

A comprehensive search on online databases was conducted from inception to May 2025, and relevant study data were extracted. The meta-analysis utilized odds ratio (OR) with 95% confidence interval (CI) as effect size measures. Subgroup analyses, heterogeneity assessment, publication bias, and sensitivity analyses were performed to ensure robustness. All statistical analyses were conducted using STATA.

**Results:**

The pooled analysis of 17 observational studies showed a significant association between the highest FFs and UPFs consumption and increased breast cancer risk (OR 1.25, 95% CI [1.09–1.43], *p* = 0.001). Subgroup analysis revealed a significant positive association between FFs and UPFs consumption and breast cancer risk in case–control studies, but not in cohort studies or menopausal status and a significant association was observed in studies with sample sizes > 1000 and < 1000. Furthermore, the association was significant in Latin America when BMI adjustment was considered for 'yes' and 'no'.

**Conclusions:**

This meta-analysis identified a significant association between the consumption of FFs and UPFs and an increased risk of breast cancer, with high intake linked to a 25% greater risk. These findings suggest that diets high in UPFs may play a role in breast cancer development. As UPF consumption continues to rise, public health strategies and regulatory policies targeting food processing, marketing, labeling, and accessibility are essential for cancer risk reduction and prevention.

**Supplementary Information:**

The online version contains supplementary material available at 10.1186/s41256-025-00425-x.

## Introduction

Breast cancer is one of the most frequently diagnosed cancers worldwide and remains a leading cause of mortality, with its incidence steadily increasing [1]. The development of breast cancer is associated with a variety of risk factors, including genetic susceptibility, positive family history, environmental factors, reproductive history, and exogenous hormone intake [2]. It is noted that 20–30% of breast cancers can be attributed to modifiable factors such as alcohol intake, obesity, physical inactivity, and unhealthy diet [3]. Numerous studies have investigated the impact of different dietary patterns on breast cancer risk. The protective effects of the Mediterranean diet on breast cancer have been consistently shown in the literature [4–6]; whereas an increased risk of breast cancer has been associated with the Western diet, which is characterized by high intakes of processed foods, red meat, and animal products [5, 7]. Over the last decade, diets in many countries have shifted towards a dramatic increase in the consumption of fast foods (FFs) and ultra-processed foods (UPFs).

A processed food dietary pattern includes FFs, alcoholic and sugar-sweetened beverages, and packaged snacks, which contribute to 25–50% of total energy intake in high and middle-income countries [8]. While UPFs are typically affordable, accessible, and energy-dense, they are also heavily modified with added sugars, salts, and saturated fats and lack essential micronutrients, fiber, protein, vitamins, and bioactive compounds [9–11]. These products also undergo a series of industrial processes, such as extrusion and molding, and are enhanced with additives, like stabilizers and preservatives, to improve the texture, appearance, and durability of foods and prevent the proliferation of microorganisms [12]. Numerous additives in processed foods, such as heterocyclic amines, aromatic polycyclic hydrocarbons, bisphenol, sodium nitrites, and titanium dioxide [13, 14], are linked to DNA damage, chronic inflammation, and endocrine disruption, all of which play roles in cancer development [15–20].

A growing body of evidence has investigated the role of UPFs consumption and cancer incidence. In a study conducted in Spain, Isaksen et al. [21] reported that a 10% increase in the proportion of UPFs in the diet was associated with a 10% increase in overall cancer [21]. In line with this study, findings by Fiolet et al. indicated a direct link between UPFs consumption and elevated risks of overall cancer [22]. In the United States, Chandran et al. found that a higher intake of energy-dense and FFs was associated with an increased risk of cancer in women [23]. Conversely, two case–control studies conducted in Spain and Iran found no significant association between UPFs consumption and breast cancer incidence [24, 25]. To the best of our knowledge, a recent systematic review and meta-analysis by Shu et al. investigated the association between UPFs and breast cancer risk, which observed a 5% increase in breast cancer incidence with each 10% rise in UPFs consumption; however, this association was not supported in cohort studies and sample sizes greater than 5,000. Additionally, the mentioned study included only six studies, limiting the strength of evidence for a definitive association [26].

Although the potential association between processed food consumption and cancer risk has been investigated in several studies, previous meta-analyses have included limited studies. Additionally, they often combined different cancer types, making it challenging to evaluate breast cancer-specific associations. Consequently, this comprehensive systematic review and meta-analysis of observational studies was conducted to address the discrepancies between the studies with the specific aim of clarifying the association between FFs and UPFs consumption and the incidence of breast cancer.

## Methods

### Study design

This systematic review and meta-analysis was conducted in accordance with the principles outlined in the Preferred Reporting Items for Systematic Reviews and Meta-Analyses (PRISMA) guidelines [27]. The aim was to assess the association between FFs and UPFs consumption with the risk of breast cancer in women.

### Search strategy

A thorough literature search was conducted using three major databases: MEDLINE/PubMed, ISI Web of Science, and Scopus, covering all available records up to May 1, 2025. The search was explicitly designed to identify observational studies investigating the relationship between the consumption of FFs and UPFs and the incidence of breast cancer. The search utilized a combination of MeSH terms and relevant keywords, such as (“ultra-processed foods” OR “processed food” OR “processed foods” OR “fast food” OR “fast foods” OR “junk food” OR “junk foods” OR “quick-service foods” OR “convenience foods” OR “takeaway foods” OR “ready-to-eat foods”) AND (“Breast cancer” OR “breast malignancy” OR “breast tumor” OR “breast malignant” OR “breast neoplasm” OR “breast carcinoma” OR “breast adenocarcinoma”). Additionally, to ensure the inclusion of all relevant studies, the reference lists of selected articles were manually reviewed for any additional sources not captured in the initial database search. The search process applied no language or publication date restrictions, ensuring a comprehensive and inclusive approach.

### Eligibility criteria

Two independent researchers (M.A. and E.H.) systematically screened titles and abstracts from the identified articles using EndNote software. Full-text versions of potentially eligible studies were retrieved and assessed based on predefined inclusion and exclusion criteria. Studies were included if they were observational, investigated FFs and UPFs consumption as the exposure, reported breast cancer incidence as the outcome, and provided sufficient data to calculate effect estimates. Exclusion criteria ruled out experimental studies, animal or in vitro research, clinical trials, reviews, editorials, and conference abstracts. Any disagreements during the selection process were resolved through discussion or, when necessary, by consultation with a third researcher (M.K.)

### Data extraction

Two authors (M.A. and E.H.) independently extracted data using a standardized data collection framework to ensure consistency. The extracted details included the first author’s last name, publication year, study location, study design, sample size, number of breast cancer cases, participants’ age range, outcomes, comparison groups, adjustments for confounding factors, and reported effect size measures including odds ratio (OR), relative risk (RR) or hazard ratio (HR) with 95% confidence intervals (CI). Any disagreements between the researchers were resolved through group discussions, with input from a third researcher (M.K.) to reach a consensus.

### Risk of bias assessment

The Newcastle–Ottawa Scale (NOS) was used to evaluate the quality of the included cohort studies [28]. This scale allocates a maximum of 9 points across three key domains: (1) the selection of study groups (up to four points), (2) the comparability of groups (up to two points), and (3) the assessment of outcomes (up to three points). Studies achieving a total NOS score of 7 or higher were categorized as high-quality.

### Statistical analysis

In the meta-analysis, OR and 95% CI were used as effect size measures, with RR and HR treated as equivalent to OR when reported [29]. Effect sizes were determined by comparing the consumption of the highest and lowest categories of FFs and UPFs. Fixed-effects models were applied to explore potential associations, while heterogeneity among studies was evaluated using the Q and I^2^ statistics, with I^2^ values exceeding 50%, indicating substantial heterogeneity [30]. Publication bias was assessed through visual inspection of funnel plots and Egger’s and Begg’s tests [31, 32], and the trim-and-fill method was used to adjust for bias if detected [33]. Subgroup analyses were performed based on study design, menopausal status, sample size, geographic location, and BMI adjustment. Sensitivity analyses were also conducted by systematically excluding one study at a time to evaluate its influence on the overall results. All statistical analyses were performed using STATA version 17.0 (Stata Corp. LLC), and a P-value of less than 0.05 was considered statistically significant.

## Results

### Study selection

**Fig. 1:**
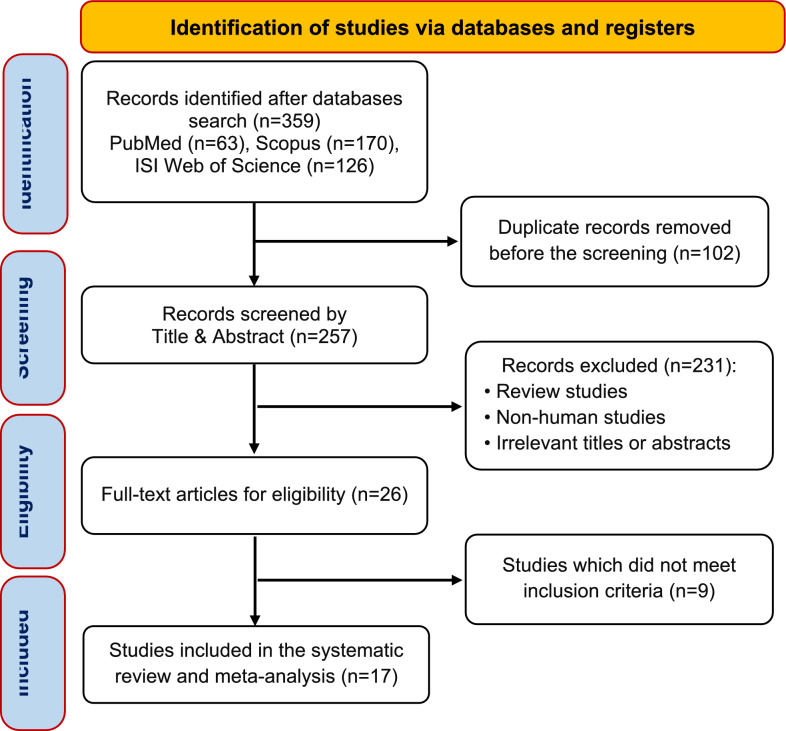
PRISMA Flow chart of the study selection process in the systematic review

We identified a total of 359 records in the initial search. After removing 102 duplicates and excluding 231 publications during the title and abstract screening, we reviewed 26 articles in full text. Of these, nine articles were eliminated for not meeting the eligibility criteria. Ultimately, 17 studies were included in the final analysis. A detailed flowchart of the search strategy is illustrated in Fig. 1.

### Study characteristics

The basic characteristics of the included studies are presented in Table 1**.** Data were collected from 17 eligible observational studies, including six cohorts and 11 case–control studies [22–24, 34–47]. These studies involved 744,277 participants, out of which 20,351 were diagnosed with breast cancer. The follow-up period for the cohort studies ranged from 5 to 22 years. The studies were published between the years 2014 and 2024. They were conducted in the following locations: the United States, South Africa, South Korea, Iran, France, Poland, England, Scotland, Wales, and six Latin American countries. Two studies were performed in postmenopausal women exclusively [37, 42], one study according to menopausal status [34], and 14 studies combined premenopausal and postmenopausal breast cancer subjects [22–24, 35, 36, 38–41, 43–47]. All included studies utilized the NOVA food classification system to classify UPFs.

**Table 1:** Basic characteristics of included studies in the meta-analysis

Study	Country	Study design	Age (y)	Sample Size	Cases	Exposure	Effect size (95% CI)	Adjustment
Nouri et al. [24]	Iran	Case–control	30–65	399	133	Ultra-processed foods	1.80 (0.92 – 3.51)	BMI, marriage age, age at the first pregnancy, breastfeeding time, fiber intake, menopausal status, Hx of abortion, family Hx of cancer and BC, wearing, vitamin D and omega-3 supplements, herbal drugs
Omofuma et al. [35]	USA	Cohort	55–74	27,464	1592	Advanced glycation end-products (AGEs)	1.30 (1.04 – 1.62)	Dietary intake of total fat and red meat, age (age at menarche, menopause, first birth), energy intake, alcohol, BMI, vigorous activity, race, marital status, education, smoking status, family Hx of BC, parity, PMH use, OC use, oophorectomy, hysterectomy
Stasiewicz et al. [39]	Poland	Case–control	40–79.9	420	190	Highly processed food, including fast foods, sweets, instant soups	0.70 (0.45 – 1.09)	age, socioeconomic status, smoking, age at menarche, menopausal status, gravidity, OC use, HRT use, family Hx of BC, chronic diseases, vitamin/mineral supplements use, BMI, WC, food consumption, PA, breastfeeding
Chang et al. [34]	England, Scotland, Wales	Cohort	40–69	197,426	1856	Ultra-processed foods	1.04 (0.91 – 1.18)	age, ethnicity, smoking status, PA, average household income, education, alcohol intake, BMI, total daily energy intake, height, family Hx of BC, index of multiple deprivation quintile, geographical region, OC use, HRT use, parity
Jacobs et al. [36]	South Africa	Case–control	> 18	792	396	Ultra-processed foods	1.03 (0.72 – 1.45)	NR
Socha et al. [37]	Poland	Case–control	NR	435	210	Smoked products	4.14 (1.30 – 13.18)	age, family Hx of BC, residence location, education, occupation, stress, age (at menarche, menopause, first birth), parity, breastfeeding duration, HRT, BMI, alcohol intake, PA
Jacobs et al. [36]	South Africa	Case–control	26–88	792	396	Processed meat	0.9 (0.61 – 1.35)	menopausal status, WC, OC use, ever breast-feeding, PA, education, income, ethnicity, age (at menarche, menopause onset), family Hx of BC
Romieu et al. [40]	Chil, Colombia, Casta Rica, Mexico	Case–control	20–45	1050	525	Meat, industrial yogurt and dairy-based drinks, industrial cheese,	1.93 (1.11 – 3.35)	full-term pregnancy, breastfeeding ever, BMI, total energy intake
Huybrechts et al. [38]	Latin America	Case–Control	20–45	406	203	Ultra-processed foods	1.93 (1.23 – 3.04)	Potential confounding factors,
Peterson et al. [42]	USA	Cohort	NR	183,548	9851	Advanced glycation end-products (AGEs)	1.00 (0.90 – 1.11)	age, study, education
Jacobs et al. [43]	South Africa	Case–control	26–88	792	396	Processed meat	0·9 (0.61 – 1.35)	menopausal status, WC, OC use, ever breast-feeding, PA, education, income, ethnicity, age at menarche, age of menopause onset, family Hx of BC
Fiolet et al. [22]	France	Cohort	18.0–72.8	82,159	739	Ultra-processed foods	1.11 (1.01 – 1.21)	age, sex, energy intake without alcohol, number of 24-h dietary records, smoking status, educational level, PA, height, BMI, alcohol intake, family Hx of cancers, menopausal status, HRT, OC, number of children, intakes of lipids, sodium, carbohydrates and Western diets
Queiroz et al. [44]	Brazil	Case–control	NR	118	59	Ultra-processed foods	2.35 (1.08 – 5.12)	Weight, excessive caloric intake
Kim et al. [45]	South Korea	Cohort	> 30	5046	72	Fast foods	1.16 (0.70 – 1.90)	age, BMI, family Hx of BC, smoking status, alcohol consumption, PA, age at menarche, parity, oral contraceptive use, Hx of BBD, HRT, menopausal status, and age at menopause
Harris et al. [46]	USA	Cohort	NR	45,204	1477	Fast foods	0.99 (0.84 – 1.17)	NR
Chandran et al. [23]	USA	Case–control	20–75	1692	803	Fast foods	1.55 (1.21 – 1.99)	age, ethnicity, country of origin, education, age at menarche, menopausal status, parity, age at first birth, breastfeeding status, family Hx of BC, HRT, OC use, Hx of BBD, study site, BMI, total MET hours per week, total energy intake
Laamiri et al. [47]	Morocco	Case–control	22–75	800	400	Processed meat	9.78 (4.37 – 20.24)	Age

(*NR*: not reported, *CI*: confidence interval, *Hx*: medical history, *BC*: breast cancer, *BBD*: benign breast disease, *PA*: physical activity, *BMI*: body mass index, *WC*: waist circumference, *HRT*: hormone replacement therapy, *OC*: oral contraceptive, *MET*: moderate-intensity aerobic exercise)

### Meta-analysis of FFs and UPFs consumption and breast cancer risk

**Fig. 2:**
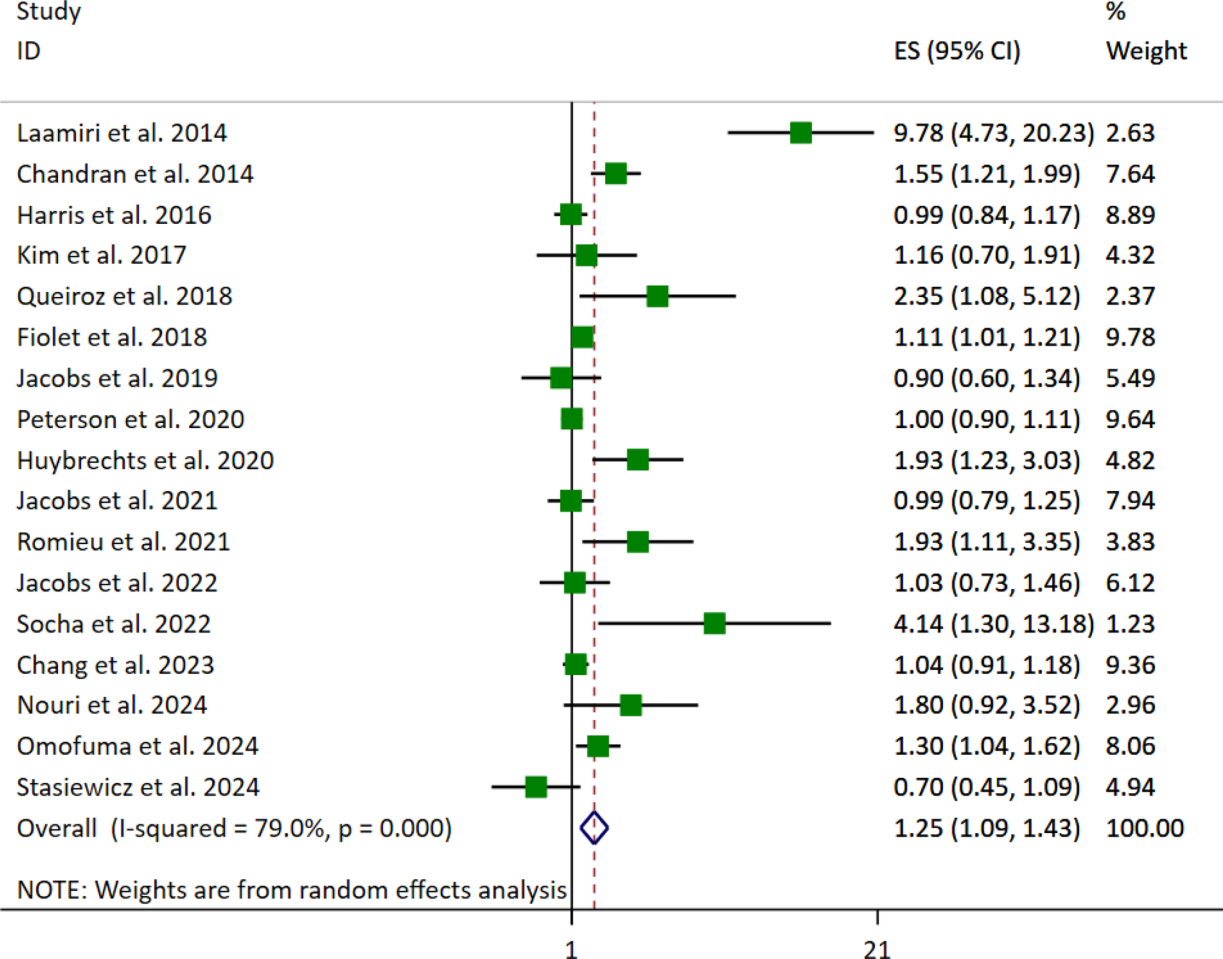
Forest plot detailing the effect size (ES) with 95% confidence intervals (CI) for the association between the highest vs. lowest fast foods (FFs) and ultra-processed foods (UPFs) intake and the risk of breast cancer in adult women

A total of 17 studies, comprising 744,277 participants and 20,351 cases of breast cancer, were included to evaluate the association between FFs and UPFs consumption and breast cancer risk. A random-effects model was applied to account for the substantial heterogeneity among studies (I^2^ = 79%, *p* < 0.001). The meta-analysis revealed a significant association between the highest versus lowest UPFs consumption categories and an increased risk of breast cancer (OR: 1.25, 95% CI [1.09—1.43], *p* = 0.001) (Table 2, Fig. 2).

**Table 2:** Meta-analyses’ findings of the association between fast foods (FFs) and ultra-processed foods (UPFs) and risk of breast cancer in women

	No. of ES		(95% CI)	p-value	I^2^	Pooled ES
Overall	17		(1.09, 1.43)	0.001*	79%	1.25
*Subgroup* *analysis* *based* *on* *the* *Study* *design*						
Case–control	11		(1.16, 2.24)	0.004*	83.7%	1.61
Cohort	6		(1.00, 1.14)	0.060	20.5%	1.06
*Subgroup* *analysis* *based* *on* *the* *Menopausal* *status*						
Premenopausal	9		(0.85, 1.30)	0.662	65.4%	1.05
Postmenopausal	10		(0.98, 1.11)	0.200	10.3%	1.04
*Subgroup* *analysis* *based* *on* *the* *Sample* *size*						
> 1000	8		(1.03, 1.15)	0.017*	62.8%	1.09
< 1000	9		(1.04, 1.38)	0.021*	85.7	1.19
*Subgroup* *analysis* *based* *on* *the* *Geographical* *region* *and* *population*						
America	4		(0.96, 1.40)	0.122	78.4%	1.16
Asia	2		(0.90, 2.06)	0.144	5.9%	1.36
Latin America	3		(1.45, 2.75)	< 0.001*	0%	2.00
Africa	4		(0.82, 3.04)	0.173	91.8%	1.58
Europe	4		(0.87, 1.27)	0.605	68.6%	1.05
*Subgroup* *analysis* *based* *on* *the* *BMI-adjusted*						
Yes	9	1.23	(1.05, 1.44)	0.010*	66.9%	0.002
No	8	1.34	(1.03, 1.73)	0.029*	85.8%	< 0.001

(*: significant, *ES*: effect size, *BMI*: body mass index)

### Subgroup analysis

**Fig. 3:**
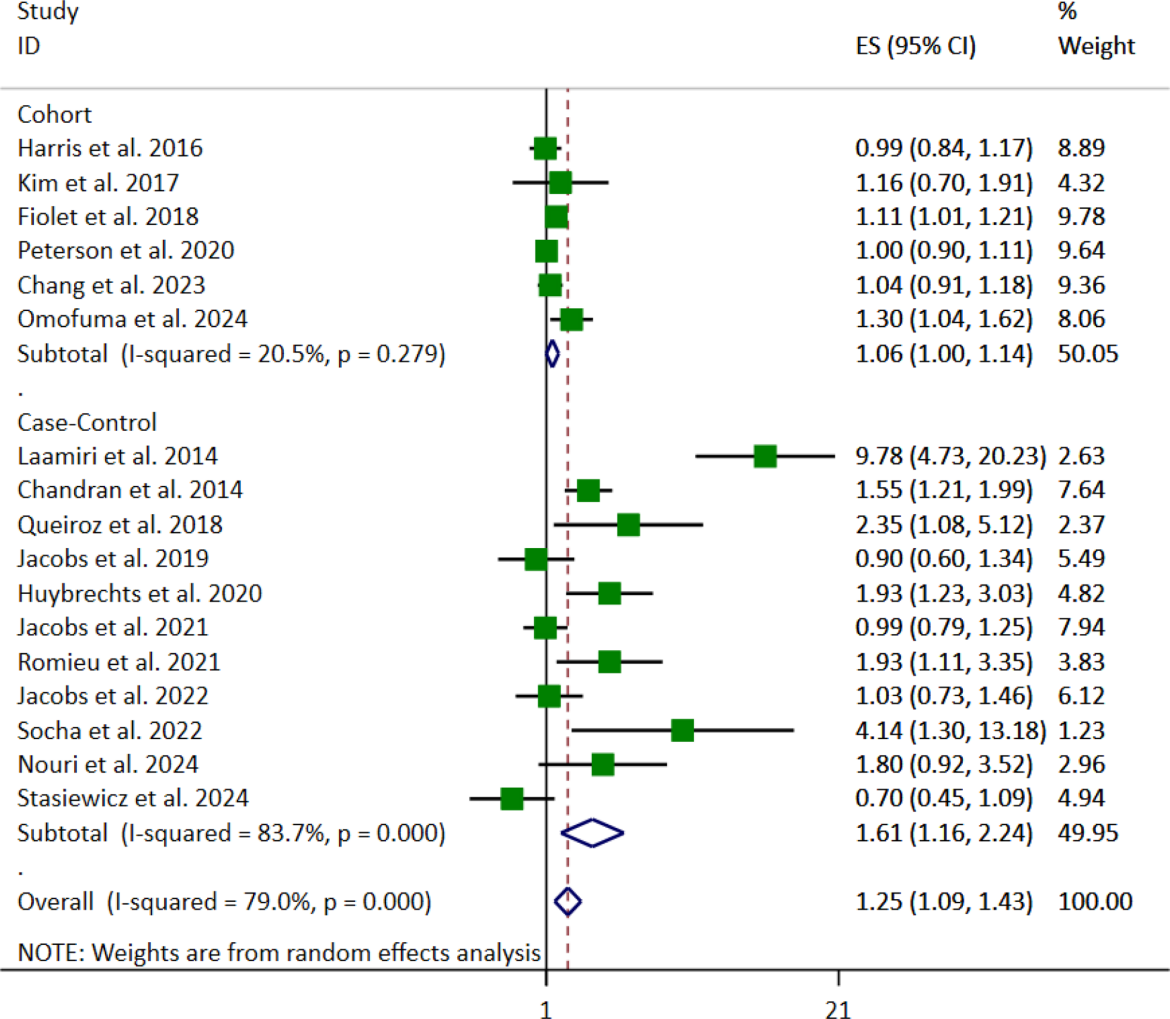
Forest plot detailing the effect size (ES) with 95% confidence intervals (CI) of the subgroup analysis based on the study design for the association between the highest vs. lowest fast foods (FFs) and ultra-processed foods (UPFs) intake and the risk of breast cancer in postmenopausal women

As presented in Table 2, subgroup analysis was performed based on study design, menopausal status, total sample size, location, and BMI adjustment. According to the study designs of the included articles, the results indicated a positive association between UPFs consumption and breast cancer risk in case–control studies (OR 1.61; 95% CI [1.16–2.24], *p* = 0.004). However, no association was observed between UPFs consumption and the risk of breast cancer in cohort studies (OR 1.06, 95% CI [1.00–1.14], *p* = 0.060) (Fig. 3).

**Fig. 4:**
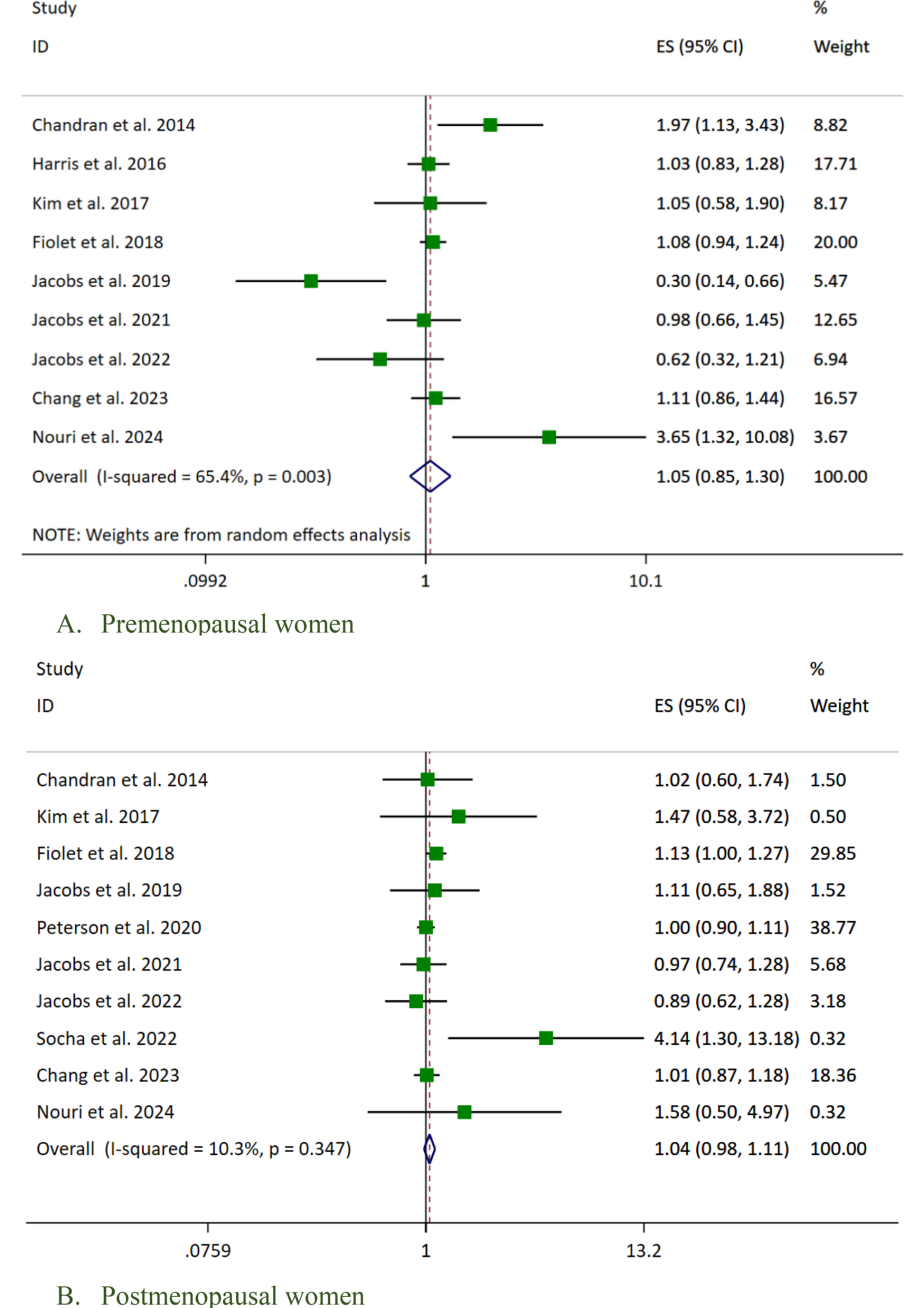
Forest plot detailing the effect size (ES) with 95% confidence intervals (CI) for the association between the highest vs. lowest fast foods (FFs) and ultra-processed foods (UPFs) intake and the risk of breast cancer in **A** premenopausal and **B** postmenopausal women

Subgroup analysis according to menopausal status showed no association between UPFs consumption and breast cancer risk in premenopausal (OR 1.05; 95% CI [0.85–1.30], *p* = 0.662) (Fig. 4A) and postmenopausal women (OR 1.04, 95% CI [0.98–1.11], *p* = 0.20) (Fig. 4B).

**Fig. 5:**
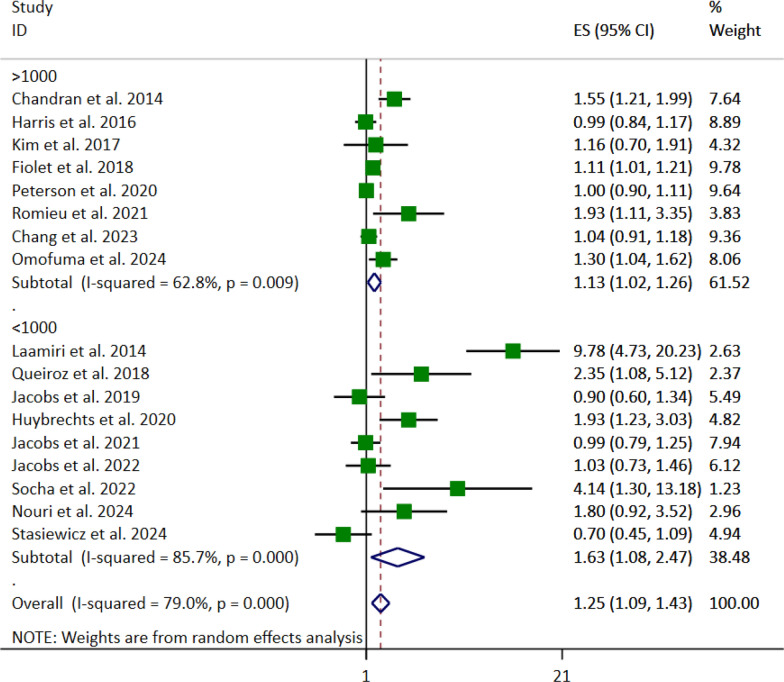
Forest plot detailing the effect size (ES) with 95% confidence intervals (CI) of the subgroup analysis based on the sample size for the association between the highest vs. lowest fast foods (FFs) and ultra-processed foods (UPFs) intake and the risk of breast cancer in women

Subgroup analysis based on the sample sizes, in both sample sizes > 1000 (OR: 1.09; 95% CI: [1.03—1.15], *p* = 0.017) and < 1000 (OR: 1.19, 95% CI: [1.04, 1.38], *p* = 0.021), the association between UPFs consumption and breast cancer risk was significant (Fig. 5).

**Fig. 6:**
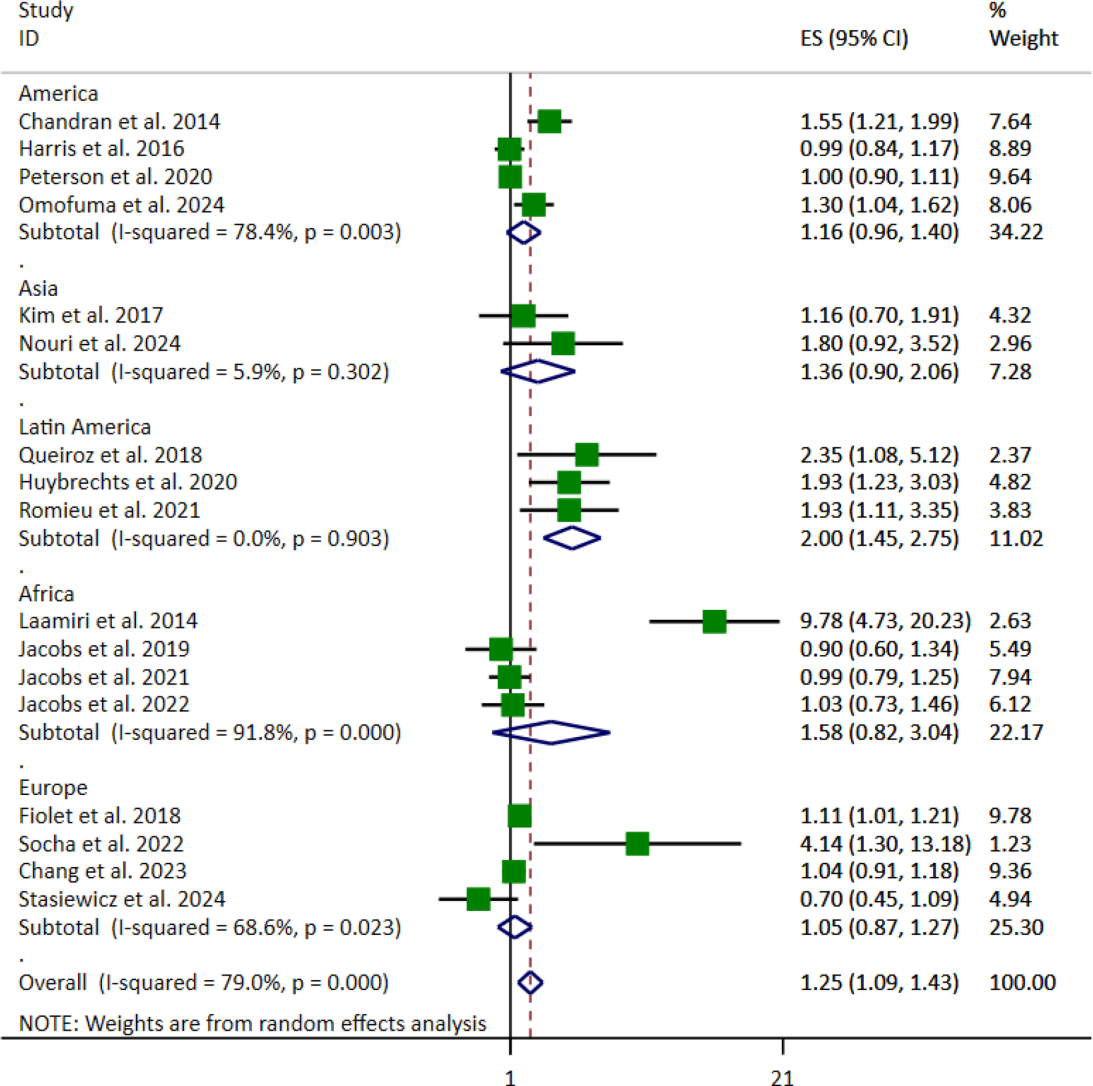
Forest plot detailing the effect size (ES) with 95% confidence intervals (CI) of the subgroup analysis based on the study location for the association between the highest vs. lowest fast foods (FFs) and ultra-processed foods (UPFs) intake and the risk of breast cancer in adult women

**Fig. 7:**
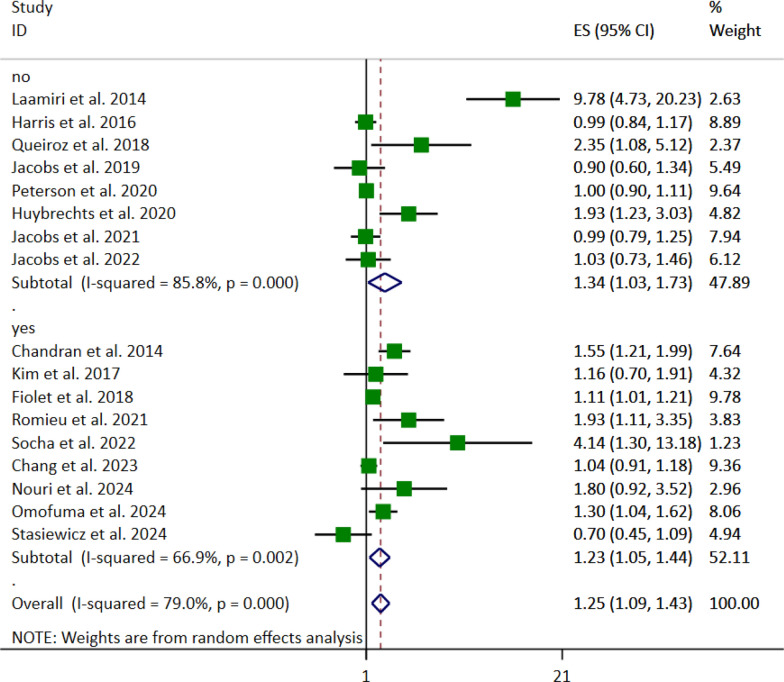
Forest plot detailing the effect size (ES) with 95% confidence intervals (CI) of the subgroup analysis based on the body mass index (BMI) for the association between the highest vs. lowest fast foods (FFs) and ultra-processed foods (UPFs) intake and the risk of breast cancer in adult women

Subgroup analysis based on location revealed the association between FFs and UPFs and breast cancer risk in Latin America (OR 2.00, 95% CI [1.45, 2.75], *p* < 0.001) (Fig. 6). However, no significant association was found in other regions. Moreover, when considering BMI adjustment, the pooled OR was (OR: 1.23, 95% CI: [1.05–1.44], *p* < 0.010) for yes and (OR 1.34, 95% CI [1.03–1.73], *p* < 0.029) for no (Fig. 7).

### Sensitivity analysis

The sensitivity analysis revealed that the overall effect size did not change after eliminating any studies (CI: 1.04–1.53) (Supplementary Fig. 1).

### Publication bias

**Fig. 8:**
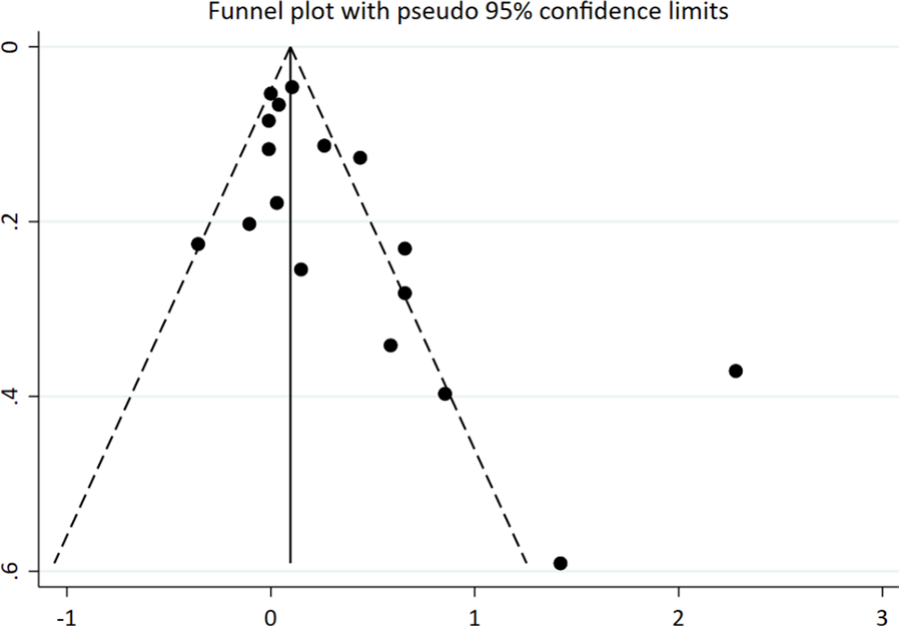
Funnel plot of publication bias with 95% confidence limits

According to funnel plots, an asymmetry was observed. In addition, Egger’s (*p* = 0.019) and Begg’s (*p* = 0.029) tests for publication bias were statistically significant. We utilized the trim and fill method to assess the missing articles; no missing article was found after meta-trim analysis (Fig. 8).

### Quality assessment

According to the Newcastle–Ottawa Scale (NOS), the quality assessment of included cohort studies (Table 3) revealed that the overall scores ranged from 6 to 9, with Harris et al. (2016) scoring the highest (9), indicating high methodological quality, while Peterson et al. (2020) received the lowest score (6). Most cohort studies met key quality criteria, including representativeness, ascertainment of exposure, and outcome assessment; however, some studies lacked sufficient follow-up duration or adequate follow-up rates. For case–control studies (Table 4), the overall quality scores ranged from 7 to 9, with Stasiewicz et al. (2024) and Chandran et al. (2014) achieving the highest scores (9). These studies generally demonstrated substantial comparability, exposure assessment, and case–control definitions, but some studies showed limitations in controls’ selection or comparability. Both tables highlight a generally high methodological quality across the included studies, ensuring robust data for further analysis.

**Table 3:** Quality assessment of the included cohort studies based on the Newcastle–Ottawa grading scale (NOS)

Cohort studies	Q1	Q2	Q3	Q4	Q5	Q6	Q7	Q8	Q9	Overall
Omofuma et al. 2024	*	*	*	*	*		*	*	*	8
Chang et al. 2023	*	*	*	*	*	*	*	–	–	7
Peterson et al. 2020	–	*	*	*	*		*	*	–	6
Fiolet et al., 2018	*	*	*	*	*	*	*	–	*	8
Kim et al. 2017	*	*	*	*	*	*	*	–	–	7
Harris et al. 2016	*	*	*	*	*	*	*	*	*	9

* = award of a star (met criterion); No star = did not meet that item

Q1: Representativeness of the exposed cohort, Q2: Selection of the non-exposed cohort, Q3: Ascertainment of exposure, Q4: Demonstration that the outcome of interest was not present at the start of the study, Q5: Comparability of cohorts based on the design or analysis, Q6: Study controls for any additional factor, Q7: Assessment of outcome, Q8: Was follow-up long enough for outcomes to occur (> 10 years), Q9: Adequacy of follow-up of cohorts (loss-to-follow-up < 20%)

**Table 4:** Quality assessment of the included case–control studies, based on the Newcastle–Ottawa grading scale (NOS)

Case–control studies	Q1	Q2	Q3	Q4	Q5	Q6	Q7	Q8	Overall
Nouri et al. [24]	*	*	*	*	*	*	*	*	8
Stasiewicz et al. [39]	*	*	*	*	**	*	*	*	9
Jacobs et al. [36]	*	*	*	*	–	*	*	*	7
Socha et al. [37]	*	*	*	*	*	*	*	*	8
Jacobs et al. [41]	*	*	*	*	–	*	*	*	7
Romieu et al. [40]	*	*	*	–	**	*	*	*	8
Huybrechts et al. [38]	*	*	*	*	*	*	*	*	8
Jacobs et al. [43]	*	*	*	*	–	*	*	*	7
Queiroz et al. [44]	*	*	*	*	*	*	*	*	8
Chandran et al. [23]	*	*	*	*	**	*	*	*	9
Laamiri et al. [47]	*	*	*	*	–	*	*	*	7

* = award of a star (met criterion); No star = did not meet that item

Q1: Is the case definition adequate?, Q2: Representativeness of the cases, Q3: Selection of controls, Q4: Definition of controls, Q5: Comparability of cases and controls based on the design or analysis, Q6: Assessment of exposure, Q7: Same method of assessment for cases and controls, Q8: Non response rate

## Discussion

In recent decades, the rising consumption of FFs and UPFs due to shifting lifestyles and evolving food cultures has become a global concern. Prior studies have identified the presence of carcinogenic chemicals in these foods, sparking interest in their potential role in cancer development. However, the evidence linking these food consumption to breast cancer risk remains inconclusive. To address this gap, our comprehensive systematic review and meta-analysis examined the relationship between FFs and UPFs consumption and breast cancer risk. The results demonstrated a significant positive association, with high UPFs consumption linked to a 25% increased risk of breast cancer compared to low consumption levels, underscoring the need for further research and public health interventions.

Our findings are consistent with previous studies. A meta-analysis by Shu L. et al. [26] on only six articles involving 462,292 participants reported a 5% increased breast cancer risk for every 10% increment in UPFs consumption [26]. Similarly, Isaksen et al. [21] showed a positive association between a 10% higher UPFs consumption and overall cancer and breast cancer risk [21]. Whereas a less consistent association between UPFs consumption and breast cancer was reported in a population-based case–control study conducted in Spain, which observed only a positive association between UPFs consumption and colorectal cancer risk, while no statistically significant correlation was observed for breast and prostate cancer [25]. Moreover, higher consumption of UPFs was not associated with an increase in breast cancer risk among South African women [36]. While these mixed findings suggest the need for further research into the role of UPFs in breast cancer across diverse populations, the large sample size and comprehensive nature of our meta-analysis provide the most robust and updated evidence addressing this research question.

Although the association between UPFs consumption and breast cancer risk remains inconclusive, several mechanisms potentially underlying breast cancer development have been proposed. UPFs are typically high in saturated fats, sugars, salt, and additives, which have been linked to an increased risk of overweight and obesity [48], an established risk factor for developing breast cancer [49]. Additionally, increased consumption of carbohydrates and a higher glycemic load have been associated with an elevated risk of breast cancer. Dietary fats can influence carcinogenesis by modulating intracellular signaling pathways [50]. Furthermore, this dietary pattern lacks essential micronutrients and bioactive compounds and is linked to lower consumption of unprocessed or healthy foods [51]. The protective effects of healthy dietary patterns against breast cancer risk were mentioned in several studies [52]. This pattern, characterized by high consumption of vegetables, fruits, low-fat products, legumes, and unprocessed foods, was associated with a 75% reduction in breast cancer risk in a study conducted by Karimi et al. [53]. The protective effect is likely attributable to polyphenolic compounds, fiber, and numerous bioactive agents that exhibit antioxidant, anti-inflammatory, antiproliferative, and apoptotic effects and lower estrogen levels, which contribute to its potential anticancer properties [54–56].

Furthermore, FFs and UPFs often undergo different transformations during industrial processing to produce the final product. Various food contaminants generated during the processing, such as trans-fats, acrylamide, heterocyclic amines, polycyclic aromatic hydrocarbons, oxyhalides, and haloacetic acids, have been associated with an increased risk of cancer in prior studies [57, 58]. Additionally, indirect contaminants produced during packaging processes, including Di(ethylhexyl) phthalate (DEHP) and bisphenol-A (BPA), have been shown to contribute to endocrine disruption, DNA damage, and cancer development [57, 59]. Another point to consider is the extensive use of food additives such as sodium nitrate and titanium dioxide (TiO2), which are commonly employed as preservatives to improve texture and have been associated with potential carcinogenic effects, as suggested by previous evidence [60].

Our analysis revealed substantial heterogeneity among studies examining the association between UPFs consumption and breast cancer risk (I^2^ = 79%, *p* = 0.001). Therefore, subgroup analysis of study design (cohort/case–control studies), menopausal status (pre-menopausal/post-menopausal breast cancer), region (America/Latin America/ Asia/ Europe/ Africa), sample size (< 1000; > 1000), and BMI adjustment were carried out to explore the source of heterogenicity. The result demonstrated that case–control studies report a stronger association, but with high heterogeneity; this discrepancy may be attributed to inherent methodological differences rather than an actual effect. Case–control studies are more susceptible to recall bias and selection bias. In contrast, cohort studies are more prone to dietary misclassification and changes in nutritional habits over extended follow-up. Similar discrepancies between study designs have been noted in previous epidemiological studies [61, 62].

No significant associations were observed when stratified by menopausal status, consistent with previous meta-analyses examining the relationship between UPFs, sweet beverage intake, and breast cancer risk [26, 63]. Furthermore, Stronger associations were found in studies with smaller sample sizes (< 1000 participants), which may reflect the implementation of more rigorous methodologies in such studies. The strongest association in different geographical regions was found in Latin America and Africa. This variable level of association in other areas is mainly due to differences in dietary patterns, UPFs definition, or genetic susceptibility. Another point to consider is the limited screening programs in these low or middle-income countries (LMICs), which often lead to more advanced and aggressive cancer diagnoses linked to modifiable risk factors like diet [64]. Additionally, underreporting of early-stage cases could strengthen the observed association [65]. Finally, as healthcare systems in LMICs improve, rising breast cancer diagnoses will lead to a more accurate understanding of the association between breast cancer risk and UPFs consumption [66]. Furthermore, adjusting for BMI weakened the observed association, suggesting that BMI may act as a mediating factor in the development of breast cancer.

### Strengths and limitations

This study has several notable strengths. One key advantage is the inclusion of a large number of observational studies compared to prior research [21, 26], allowing for precise subgroup meta-analyses based on various factors and providing sufficient statistical power to explore multiple potential risk factors. Additionally, the included studies accounted for several confounding variables, and no evidence of publication bias was observed in funnel plots or Begg’s and Egger’s tests. Nonetheless, some limitations must be acknowledged. Case–control studies in this meta-analysis exhibited a stronger association than cohort studies, with high levels of heterogeneity, raising the possibility of recall and selection biases in retrospective designs. In contrast, cohort studies, which tend to be less susceptible to recall bias, showed a weaker association. This discrepancy highlights the need for more prospective studies to confirm these findings. While most studies adjusted for a wide range of confounders, residual confounding cannot be entirely excluded. A high level of heterogeneity was also identified, which was thoroughly investigated through subgroup analyses. However, the exact sources of this heterogeneity remain unclear, which may be attributed to differences in study design, dietary assessment methods, regional dietary patterns, and adjustment for confounders. Finally, the absence of local and hospital-based clinical data on tumor characteristics remains a limitation to fully address whether the observed associations reflect actual dietary risks or are influenced by disparities in the healthcare system and access to screening programs.

## Conclusions

This meta-analysis revealed a significant positive association between FFs and UPFs consumption and breast cancer risk, with high UPFs intake linked to a 25% increased risk compared to low intake. The findings suggest that diets high in UPFs may contribute to breast cancer development and potentially other non-communicable diseases. As UPFs become more prevalent in modern diets, the study emphasizes the need for policy actions to reduce their negative health impacts. These include stricter regulations on the industrial processing and marketing of UPFs, as well as public health initiatives promoting awareness of their risks. Educational campaigns and clear labeling on UPFs can inform consumers and encourage healthier choices. Further research is necessary to investigate how specific risk factors, including food composition, additives, packaging materials, and contaminants in FFs and UPFs, may influence breast cancer development.

## Supplementary Information

Additional file1.

## Data Availability

All data used in this meta-analysis were extracted from published studies. The datasets supporting the findings of this study are available from the original sources, which are cited in the manuscript. Additional information can be provided by the corresponding author upon reasonable request.

## References

[1] Xu Y, Gong M, Wang Y, Yang Y, Liu S, Zeng Q. Global trends and forecasts of breast cancer incidence and deaths. Sci Data. 2023;10(1):334. 37244901 10.1038/s41597-023-02253-5 PMC10224917

[2] Clinton SK, Giovannucci EL, Hursting SD. The world cancer research fund/American institute for cancer research third expert report on diet, nutrition, physical activity, and cancer: impact and future directions. J Nutr. 2020;150(4):663–71. 31758189 10.1093/jn/nxz268 PMC7317613

[3] Marino P, Mininni M, Deiana G, Marino G, Divella R, Bochicchio I, et al. Healthy lifestyle and cancer risk: modifiable risk factors to prevent cancer. Nutrients. 2024;16(6):800. 38542712 10.3390/nu16060800 PMC10974142

[4] Zamzam S, Said S, Yaghi J, Faisal FS, Hassan D, Abdul Majeed S, et al. Dietary patterns associated with breast cancer in the middle east: a scoping review. Nutrients. 2024;16(5):579. 38474708 10.3390/nu16050579 PMC10934189

[5] Dianatinasab M, Rezaian M, HaghighatNezad E, Bagheri-Hosseinabadi Z, Amanat S, Rezaeian S, et al. Dietary patterns and risk of invasive ductal and lobular breast carcinomas: a systematic review and meta-analysis. Clin Breast Cancer. 2020;20(4):e516–28. 32362500 10.1016/j.clbc.2020.03.007

[6] Karimi M, Asbaghi O, Hooshmand F, Aghayan AH, Shariati AA, Kazemi K, et al. Adherence to mediterranean diet and breast cancer risk: a meta-analysis of prospective observational studies. Health Sci Rep. 2025;8(4): e70736. 40256142 10.1002/hsr2.70736 PMC12007187

[7] Carrera-Bastos P, Fontes-Villalba M, O’Keefe JH, Lindeberg S, Cordain L. The Western diet and lifestyle and diseases of civilization. Res Rep Clin Cardiol. 2011. 10.2147/RRCC.S16919.

[8] Monteiro CA, Moubarac JC, Cannon G, Ng SW, Popkin B. Ultra-processed products are becoming dominant in the global food system. Obes Rev. 2013;14:21–8. 24102801 10.1111/obr.12107

[9] Askari M, Heshmati J, Shahinfar H, Tripathi N, Daneshzad E. Ultra-processed food and the risk of overweight and obesity: a systematic review and meta-analysis of observational studies. Int J Obes. 2020;44(10):2080–91. 10.1038/s41366-020-00650-z 32796919

[10] Luiten CM, Steenhuis IH, Eyles H, Mhurchu CN, Waterlander WE. Ultra-processed foods have the worst nutrient profile, yet they are the most available packaged products in a sample of New Zealand supermarkets. Public Health Nutr. 2016;19(3):530–8. 26222226 10.1017/S1368980015002177 PMC10271194

[11] Machado PP, Steele EM, Levy RB, Sui Z, Rangan A, Woods J, et al. Ultra-processed foods and recommended intake levels of nutrients linked to non-communicable diseases in Australia: evidence from a nationally representative cross-sectional study. BMJ Open. 2019;9(8): e029544. 31462476 10.1136/bmjopen-2019-029544 PMC6720475

[12] Monteiro CA, Cannon G, Levy R, Moubarac J-C, Jaime P, Martins AP, et al. NOVA. The star shines bright. World Nutrition. 2016;7(1–3):28–38.

[13] Cox S, Sandall A, Smith L, Rossi M, Whelan K. Food additive emulsifiers: a review of their role in foods, legislation and classifications, presence in food supply, dietary exposure, and safety assessment. Nutr Rev. 2021;79(6):726–41. 32626902 10.1093/nutrit/nuaa038

[14] ELshreif HM, Elkhoudary M, Abdel-Salam RA, Hadad G, El-Gendy A. A review on food additives from the definition, and types to the method of analysis. Records Pharmaceut Biomed Sci. 2023;7(1):48–64.

[15] de Deus MR, Pimenta AM, Gea A, de la Fuente-Arrillaga C, Martinez-Gonzalez MA, Lopes ACS, et al. Ultraprocessed food consumption and risk of overweight and obesity: the university of Navarra follow-up (SUN) cohort study. Am J Clin Nutr. 2016;104(5):1433–40. 27733404 10.3945/ajcn.116.135004

[16] Bancil AS, Sandall AM, Rossi M, Chassaing B, Lindsay JO, Whelan K. Food additive emulsifiers and their impact on gut microbiome, permeability, and inflammation: mechanistic insights in inflammatory bowel disease. J Crohns Colitis. 2021;15(6):1068–79. 33336247 10.1093/ecco-jcc/jjaa254

[17] Warner JO. Artificial food additives: hazardous to long-term health? Arch Dis Child. 2024;109(11):882–5. 38423749 10.1136/archdischild-2023-326565

[18] Rico-Campà A, Martínez-González MA, Alvarez-Alvarez I, de Deus MR, De La Fuente-Arrillaga C, Gómez-Donoso C, et al. Association between consumption of ultra-processed foods and all cause mortality: SUN prospective cohort study. BMJ. 2019. 10.1136/bmj.l1949. 31142450 10.1136/bmj.l1949 PMC6538973

[19] Srour B, Fezeu LK, Kesse-Guyot E, Allès B, Debras C, Druesne-Pecollo N, et al. Ultraprocessed food consumption and risk of type 2 diabetes among participants of the NutriNet-Santé prospective cohort. JAMA Intern Med. 2020;180(2):283–91. 31841598 10.1001/jamainternmed.2019.5942 PMC6990737

[20] Srour B, Fezeu LK, Kesse-Guyot E, Allès B, Méjean C, Andrianasolo RM, et al. Ultra-processed food intake and risk of cardiovascular disease: prospective cohort study (NutriNet-Santé). BMJ. 2019;50:365. 10.1136/bmj.l1451 PMC6538975 31142457

[21] Isaksen IM, Dankel SN. Ultra-processed food consumption and cancer risk: a systematic review and meta-analysis. Clin Nutr. 2023;42(6):919–28. 37087831 10.1016/j.clnu.2023.03.018

[22] Fiolet T, Srour B, Sellem L, Kesse-Guyot E, Allès B, Méjean C, et al. Consumption of ultra-processed foods and cancer risk: results from NutriNet-Santé prospective cohort. BMJ. 2018;22:360. 10.1136/bmj.k322 PMC5811844 29444771

[23] Chandran U, McCann SE, Zirpoli G, Gong Z, Lin Y, Hong C-C, et al. Intake of energy-dense foods, fast foods, sugary drinks, and breast cancer risk in African American and European American women. Nutr Cancer. 2014;66(7):1187–99. 25265504 10.1080/01635581.2014.951737 PMC4201626

[24] Nouri M, Mansouri F, Jafari F, Ranjbar Zahedani M, Jalali S, Heidari Z, et al. Association between processed and ultra-processed food intake and the risk of breast cancer: a case-control study. BMC Cancer. 2024;24(1):1234. 39375621 10.1186/s12885-024-13014-x PMC11460039

[25] Romaguera D, Fernández-Barrés S, Gracia-Lavedán E, Vendrell E, Azpiri M, Ruiz-Moreno E, et al. Consumption of ultra-processed foods and drinks and colorectal, breast, and prostate cancer. Clin Nutr. 2021;40(4):1537–45. 33743289 10.1016/j.clnu.2021.02.033

[26] Shu L, Zhang X, Zhu Q, Lv X, Si C. Association between ultra-processed food consumption and risk of breast cancer: a systematic review and dose-response meta-analysis of observational studies. Front Nutr. 2023;10:1250361. 37731393 10.3389/fnut.2023.1250361 PMC10507475

[27] Sohrabi C, Franchi T, Mathew G, Kerwan A, Nicola M, Griffin M, et al. PRISMA 2020 statement: what’s new and the importance of reporting guidelines. Elsevier; 2021. p. 105978. 10.1016/j.ijsu.2021.105918 33789825

[28] Peterson J, Welch V, Losos M, Tugwell PJ. The Newcastle-Ottawa scale (NOS) for assessing the quality of nonrandomised studies in meta-analyses. Ottawa: Ottawa Hosp Res Inst. 2011;2(1):1–2.

[29] Symons M, Moore D. Hazard rate ratio and prospective epidemiological studies. J Clin Epidemiol. 2002;55(9):893–9. 12393077 10.1016/s0895-4356(02)00443-2

[30] Higgins JP, Thompson SG, Deeks JJ, Altman DG. Measuring inconsistency in meta-analyses. BMJ. 2003;327(7414):557–60. 12958120 10.1136/bmj.327.7414.557 PMC192859

[31] Begg CB, Mazumdar M. Operating characteristics of a rank correlation test for publication bias. Biometrics. 1994;50:1088–101. 7786990

[32] Egger M, Smith GD, Schneider M, Minder C. Bias in meta-analysis detected by a simple, graphical test. BMJ. 1997;315(7109):629–34. 9310563 10.1136/bmj.315.7109.629 PMC2127453

[33] Duval S, Tweedie R. Trim and fill: a simple funnel-plot–based method of testing and adjusting for publication bias in meta-analysis. Biometrics. 2000;56(2):455–63. 10877304 10.1111/j.0006-341x.2000.00455.x

[34] Chang K, Gunter MJ, Rauber F, Levy RB, Huybrechts I, Kliemann N, et al. Ultra-processed food consumption, cancer risk and cancer mortality: a large-scale prospective analysis within the UK Biobank. EClinicalMedicine. 2023;56:101840. 36880051 10.1016/j.eclinm.2023.101840 PMC9985039

[35] Omofuma OO, Steck SE, Olshan AF, Troester MA. The association between meat and fish intake by preparation methods and breast cancer in the Carolina breast cancer study (CBCS). Breast Cancer Res Treat. 2022;193(1):187–201. 35275284 10.1007/s10549-022-06555-x PMC8997170

[36] Jacobs I, Taljaard-Krugell C, Wicks M, Cubasch H, Joffe M, Laubscher R, et al. Degree of food processing and breast cancer risk in black urban women from Soweto, South African: the South African Breast Cancer study. Br J Nutr. 2022;128(11):2278–89. 35109954 10.1017/S0007114522000423 PMC9346100

[37] Socha M, Sobiech KA. Eating habits, risk of breast cancer, and diet-dependent quality of life in postmenopausal women after mastectomy. J Clin Med. 2022;11(15):4287. 35893378 10.3390/jcm11154287 PMC9331180

[38] Huybrechts I, Romieu I, Kandpur N, Katsikari K, Torres-Mejia G, Sanchez GI, et al. Ultra-processed food consumption and breast cancer risk. Proc Nutrit Soc. 2020;79(OCE2):E182.

[39] Stasiewicz B, Wadolowska L, Biernacki M, Slowinska MA, Stachowska E. Compliance with the WCRF/AICR recommendations in qualitative adaptation and the occurrence of breast cancer: a case-control study. Cancers. 2024;16(2):468. 38275908 10.3390/cancers16020468 PMC11154306

[40] Romieu I, Khandpur N, Katsikari A, Biessy C, Torres-Mejía G, Ángeles-Llerenas A, et al. Consumption of industrial processed foods and risk of premenopausal breast cancer among Latin American women: the PRECAMA study. BMJ Nutrit, Prevent Health. 2022;5(1):1. 10.1136/bmjnph-2021-000335 PMC9237890 35814719

[41] Jacobs I, Taljaard-Krugell C, Wicks M, Cubasch H, Joffe M, Laubscher R, et al. Dietary patterns and breast cancer risk in Black Urban South African Women: the SABC study. Nutrients. 2021;13(11):4106. 34836361 10.3390/nu13114106 PMC8617719

[42] Peterson LL, Park S, Park Y, Colditz GA, Anbardar N, Turner DP. Dietary advanced glycation end products and the risk of postmenopausal breast cancer in the National Institutes of Health-AARP diet and health study. Cancer. 2020;126(11):2648–57. 32097496 10.1002/cncr.32798 PMC7220830

[43] Jacobs I, Taljaard-Krugell C, Ricci C, Vorster H, Rinaldi S, Cubasch H, et al. Dietary intake and breast cancer risk in black South African women: the South African Breast Cancer study. Br J Nutr. 2019;121(5):591–600. 30704540 10.1017/S0007114518003744 PMC6521785

[44] Queiroz SA, de Sousa IM, de Melo Silva FR, de Oliveira LC, Fayh PT. Nutritional and environmental risk factors for breast cancer: a case-control study. Scientia Medica. 2018;28(2):2.

[45] Kim JH, Lee J, Jung S-Y, Kim J. Dietary factors and female breast cancer risk: a prospective cohort study. Nutrients. 2017;9(12):1331. 29215604 10.3390/nu9121331 PMC5748781

[46] Harris HR, Willett WC, Vaidya RL, Michels KB. Adolescent dietary patterns and premenopausal breast cancer incidence. Carcinogenesis. 2016;37(4):376–84. 26905584 10.1093/carcin/bgw023 PMC5006215

[47] Laamiri FZ, Bouayad A, Otmani A, Ahid S, Mrabet M, Barkat A. Dietery factor obesity microenvironnement and breast cancer. Gland Surg. 2014;3(3):165. 25207209 10.3978/j.issn.2227-684X.2014.06.01 PMC4139133

[48] Monda A, de Stefano MI, Villano I, Allocca S, Casillo M, Messina A, et al. Ultra-processed food intake and increased risk of obesity: a narrative review. Foods. 2024;13(16):2627. 39200554 10.3390/foods13162627 PMC11353718

[49] Picon-Ruiz M, Morata-Tarifa C, Valle-Goffin JJ, Friedman ER, Slingerland JM. Obesity and adverse breast cancer risk and outcome: mechanistic insights and strategies for intervention. CA: Cancer J Clin. 2017;67(5):378–97. 28763097 10.3322/caac.21405 PMC5591063

[50] Choi Y, Giovannucci E, Lee JE. Glycaemic index and glycaemic load in relation to risk of diabetes-related cancers: a meta-analysis. Br J Nutr. 2012;108(11):1934–47. 23167978 10.1017/S0007114512003984

[51] Lane MM, Gamage E, Du S, Ashtree DN, McGuinness AJ, Gauci S, et al. Ultra-processed food exposure and adverse health outcomes: umbrella review of epidemiological meta-analyses. BMJ. 2024. 10.1136/bmj-2023-077310. 38418082 10.1136/bmj-2023-077310 PMC10899807

[52] Dandamudi A, Tommie J, Nommsen-Rivers L, Couch S. Dietary patterns and breast cancer risk: a systematic review. Anticancer Res. 2018;38(6):3209–22. 29848668 10.21873/anticanres.12586

[53] Karimi Z, Jessri M, Houshiar-Rad A, Mirzaei H-R, Rashidkhani B. Dietary patterns and breast cancer risk among women. Public Health Nutr. 2014;17(5):1098–106. 23651876 10.1017/S1368980013001018 PMC10282447

[54] Aune D, Chan D, Greenwood D, Vieira A, Rosenblatt DN, Vieira R, et al. Dietary fiber and breast cancer risk: a systematic review and meta-analysis of prospective studies. Ann Oncol. 2012;23(6):1394–402. 22234738 10.1093/annonc/mdr589

[55] Bilal I, Chowdhury A, Davidson J, Whitehead S. Phytoestrogens and prevention of breast cancer: the contentious debate. World J Clin Oncol. 2014;5(4):705. 25302172 10.5306/wjco.v5.i4.705 PMC4129534

[56] Virani S, Afreen S, Perthiani A, Sangster E, Lanka N, Acharya P, et al. The impact of dietary unsaturated fat or the mediterranean diet on women diagnosed with breast cancer: a systematic review. Cureus. 2024. 10.7759/cureus.65362. 39184716 10.7759/cureus.65362 PMC11344571

[57] Vignesh A, Amal TC, Vasanth K. Food contaminants: Impact of food processing, challenges and mitigation strategies for food security. Food Res Int. 2024;191: 114739. 39059927 10.1016/j.foodres.2024.114739

[58] Bulanda S, Janoszka B. Consumption of thermally processed meat containing carcinogenic compounds (polycyclic aromatic hydrocarbons and heterocyclic aromatic amines) versus a risk of some cancers in humans and the possibility of reducing their formation by natural food additives-a literature review. Int J Environ Res Public Health. 2022;19(8):4781. 35457645 10.3390/ijerph19084781 PMC9024867

[59] Pandir D. DNA damage in human germ cell exposed to the some food additives in vitro. Cytotechnology. 2016;68(4):725–33. 25501537 10.1007/s10616-014-9824-y PMC4960123

[60] Chazelas E, Pierre F, Druesne-Pecollo N, Esseddik Y, Szabo de Edelenyi F, Agaesse C, et al. Nitrites and nitrates from food additives and natural sources and cancer risk: results from the NutriNet-Santé cohort. Int J Epidemiol. 2022;51(4):1106–19. 35303088 10.1093/ije/dyac046 PMC9365633

[61] Chlebowski RT, Anderson GL, Gass M, Lane DS, Aragaki AK, Kuller LH, et al. Estrogen plus progestin and breast cancer incidence and mortality in postmenopausal women. JAMA. 2010;304(15):1684–92. 20959578 10.1001/jama.2010.1500 PMC5142300

[62] Debras C, Chazelas E, Srour B, Druesne-Pecollo N, Esseddik Y, de Edelenyi FS, et al. Artificial sweeteners and cancer risk: Results from the NutriNet-Santé population-based cohort study. PLoS Med. 2022;19(3): e1003950. 35324894 10.1371/journal.pmed.1003950 PMC8946744

[63] Llaha F, Gil-Lespinard M, Unal P, de Villasante I, Castañeda J, Zamora-Ros R. Consumption of sweet beverages and cancer risk a systematic review and meta-analysis of observational studies. Nutrients. 2021;13(2):516. 33557387 10.3390/nu13020516 PMC7915548

[64] Kamaraju S, Drope J, Sankaranarayanan R, Shastri S, editors. Cancer prevention in low-resource countries: an overview of the opportunity. American Society of Clinical Oncology educational book American Society of Clinical Oncology Annual Meeting; 2020. 10.1200/EDBK_280625 PMC7935443 32239989

[65] Martei YM, Pace LE, Brock JE, Shulman LN. Breast cancer in low-and middle-income countries: why we need pathology capability to solve this challenge. Clin Lab Med. 2017;38(1):161. 29412880 10.1016/j.cll.2017.10.013 PMC6277976

[66] Lian Y, Wang G-P, Chen G-Q, Chen H-N, Zhang G-Y. Association between ultra-processed foods and risk of cancer: a systematic review and meta-analysis. Front Nutr. 2023;10:1175994. 37360305 10.3389/fnut.2023.1175994 PMC10285062

