# Supplementary material for: Consumption of fast foods and ultra-processed foods and breast cancer risk: a systematic review and meta-analysis
Source: Glob Health Res Policy. 2025 Jul 1;10:25. doi: 10.1186/s41256-025-00425-x (PMC12210537; doi:10.1186/s41256-025-00425-x)
Supplement: Supplementary file 1 — Additional file1. [file 41256_2025_425_MOESM1_ESM.docx]

**Supplementary Files**

*************************************************************************************

**Supplementary Table 1.** Search strategy

|  | | **Search terms** | |
| --- | --- | --- | --- |
| ***Exposure*** | (“ultra-processed foods” OR “processed food” OR “processed foods” OR “fast food” OR “fast foods” OR “junk food” OR “junk foods” OR “quick-service foods” OR “convenience foods” OR “takeaway foods” OR “ready-to-eat foods”) | |  |
| ***Outcomes*** | (“Breast cancer” OR “breast malignancy” OR “breast tumor” OR “breast malignant” OR “breast neoplasm” OR “breast carcinoma” OR “breast adenocarcinoma”) | |  |
| ***Study designs*** | "observational" OR "population-based" OR "epidemiological" OR “Cohort” OR "incidence" OR "cross-sectional" OR "case-control" OR "odds ratio" OR "hazard ratio" OR "risk ratio" OR "relative risk" OR "risk factors" OR "outcome assessment" | |  |

**Supplementary Table 2.** Search lines in databases (Date search: 01-May-2025)

| **Databases** | **Search lines** | **No.** |
| --- | --- | --- |
| **PubMed** | (("ultra-processed foods"[Title/Abstract] OR "processed food"[Title/Abstract] OR "processed foods"[Title/Abstract] OR "fast food"[Title/Abstract] OR "fast foods"[Title/Abstract] OR "junk food"[Title/Abstract] OR "junk foods"[Title/Abstract] OR "quick-service foods"[Title/Abstract] OR "convenience foods"[Title/Abstract] OR "takeaway foods"[Title/Abstract] OR "ready-to-eat foods"[Title/Abstract])) AND (("Breast cancer"[Title/Abstract] OR "breast malignancy"[Title/Abstract] OR "breast tumor"[Title/Abstract] OR "breast malignant"[Title/Abstract] OR "breast neoplasm"[Title/Abstract] OR "breast carcinoma"[Title/Abstract] OR "breast adenocarcinoma"[Title/Abstract])) | 63 |
| **ISI WoS** | Results for (“ultra-processed foods” OR “processed food” OR “processed foods” OR “fast food” OR “fast foods” OR “junk food” OR “junk foods” OR “quick-service foods” OR “convenience foods” OR “takeaway foods” OR “ready-to-eat foods”) (Topic) AND (“Breast cancer” OR “breast malignancy” OR “breast tumor” OR “breast malignant” OR “breast neoplasm” OR “breast carcinoma” OR “breast adenocarcinoma”) (Topic) | 126 |
| **Scopus** | ( TITLE-ABS-KEY ( ( "ultra-processed foods"  OR  "processed food"  OR  "processed foods"  OR  "fast food"  OR  "fast foods"  OR  "junk food"  OR  "junk foods"  OR  "quick-service foods"  OR  "convenience foods"  OR  "takeaway foods"  OR  "ready-to-eat foods" ) )  AND  TITLE-ABS-KEY ( ( "Breast cancer"  OR  "breast malignancy"  OR  "breast tumor"  OR  "breast malignant"  OR  "breast neoplasm"  OR  "breast carcinoma"  OR  "breast adenocarcinoma" ) ) ) | 170 |
| **All** | PubMed (63) + ISI WoS (126) + Scopus (170) = 359  Duplicates: 102  Reminded (after removal of duplicates) = 257 | **359** |


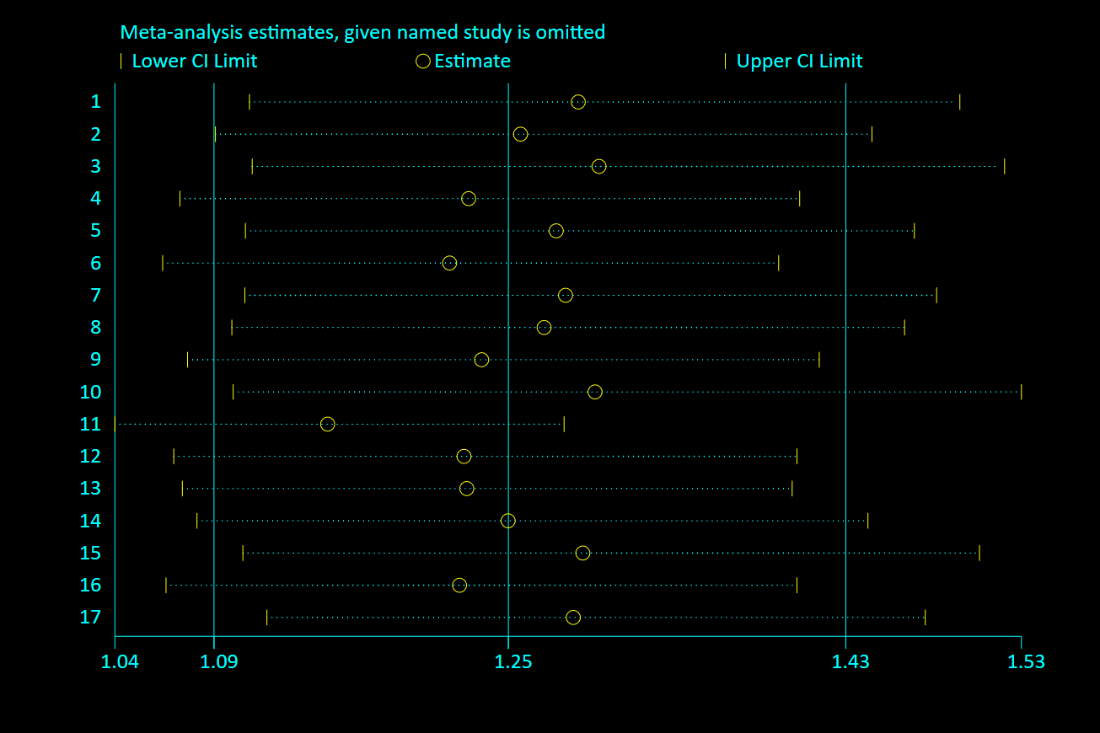


**Supplementary Figure 1.** Sensitivity analysis
